# Trends of a decade in risk factors of patient delay among pulmonary tuberculosis patients during fast aging and urbanization - analysis of surveillance data from 2008 to 2017 in Wuhan, China

**DOI:** 10.1186/s12889-023-15707-7

**Published:** 2023-05-02

**Authors:** Xiaojun Wang, Yuehua Li, Qian Fu, Meilan Zhou

**Affiliations:** 1 Wuhan Institute for Tuberculosis Control, Wuhan Pulmonary Hospital, Wuhan, China; 2 School of Medicine and Health Management, Tongji Medical College, Huazhong University of Science and Technology, No. 13 Hangkong Road, Wuhan, 430030 China

**Keywords:** Patient delay, Tuberculosis, Risk factors, Aging, Urbanization

## Abstract

**Background:**

Tuberculosis (TB) is a leading infectious cause of morbidity and mortality worldwide. However, delay in health care seeking has remained unacceptably high. The aim of this study was to clarify the trend of patient delay and its associated risk factors during rapid aging and urbanization in Wuhan, China from 2008 to 2017.

**Methods:**

A total of 63,720 TB patients registered at Wuhan TB Information Management System from January 2008 to December 2017 were included. Long patient delay (LPD) was defined as patient delay longer than 14 days. Independent associations of area and household identity with LPD, as well their interaction effect, were tested by logistic regression models.

**Results:**

Among 63,720 pulmonary TB patients, 71.3% were males, the mean age was 45.5 ± 18.8 years. The median patient delay was 10 days (IQR, 3–28). A total of 26,360 (41.3%) patients delayed for more than 14 days. The proportion of LPD decreased from 44.8% in 2008 to 38.3% in 2017. Similar trends were observed in all the subgroups by gender, age and household, except for living area. The proportion of LPD decreased from 46.3 to 32.8% in patients living near downtown and increased from 43.2 to 45.2% in patients living far from downtown. Further interaction effect analysis showed that among patients living far from downtown, the risk of LPD for local patients increased with age, while decreased with age for migrant patients.

**Conclusion:**

Although the overall LPD among pulmonary TB patients declined in the past decade, the extent of reduction varied in different subgroups. The elderly local and young migrant patients living far from downtown are the most vulnerable groups to LPD in Wuhan, China.

**Supplementary Information:**

The online version contains supplementary material available at 10.1186/s12889-023-15707-7.

## Background

Tuberculosis (TB) is a leading infectious cause of morbidity and mortality worldwide, accounting for 10 million new cases and 1.3 million deaths in 2020 [1]. The disease burden is disproportionately concentrated in low-income and middle-income countries, with over 95% TB deaths contributed by these regions [2, 3]. Many of these deaths are preventable through early diagnosis and treatment, yet, in 2019, nearly 2.9 million TB cases remained underreported or underdiagnosed globally [4]. Delay in health care seeking and treatment initiation of TB has remained unacceptably high especially among high burden countries [5].

Active TB patients may be asymptomatic or present with non-specific symptoms that resemble other clinical conditions in the early stages of the disease [6]. Failure to timely detect and treat TB could worsen illness severity, prolong patient suffering, increase the risk of patient death, and facilitate the transmission of the disease to close contacts [7, 8]. TB patients may delay to see the doctor due to reasons from various levels, such as personal demographic characteristics, health care delivery or social factors [9–11]. Being aged or migration have been proved the risk factors of long patient delay (LPD) in TB patients, thus further implies the great challenge posed by aging or urbanization on TB patient finding [12]. However, the cumulative effect of rapid urbanization and aging on TB patient delay has not been reported.

The rapid increase in migration and the elderly has brought great challenges to the detection of TB patients. Wuhan, the capital of Hubei province and the largest metropolis in central China, makes a good representative of urbanization and aging in China (Table S1). From 2008 to 2017, the resident population of Wuhan increased by 21.40%, from 8.97 to 10.89 million, with the percentage of the population aged ≥ 60 years increased by 52.15%, from 13.77–20.95% [13]. Therefore, this study analyses the 10-year surveillance data from 2008 to 2017 to clarify the trend of patient delay and its risk factors in the process of rapid aging and urbanization.

## Methods

### Study population and data collection

The data was from TB Information Management System (TBIMS) which was a unified standardized TB patient case registration system established by the Chinese Center for Disease Control and Prevention (CDC) to report and manage TB patients. All health care providers were required to report the suspected or laboratory confirmed TB cases within 24 h and refer these cases to designated TB hospitals for diagnosis and treatment. The data of TB patients were collected and entered into TBIMS by trained physicians of the designated hospitals, and checked daily by the dedicated public health physicians.

A total of 63,798 pulmonary TB patients were registered at Wuhan TBIMS from January 2008 to December 2017. Pulmonary TB patients (78, 0.1%) were excluded from the study due to missing date of onset of symptoms or the first medical visit, and eventually 63,720 pulmonary TB patients were included (Comparison between included and excluded pulmonary TB participants was showed in Table S2). The following characteristics and clinical data of patients were extracted from TBIMS: age, gender, area, ethnicity, household identity, occupation, ways of discovering patients, treatment history, bacteriology results, the date of onset of symptoms and the date of first medical visit.

### Assessment of socioeconomic regions and covariates

The municipal administration of Wuhan consists of 13 districts (Table S3, Figure S1). According to the administrative division, the city is divided into near downtown areas (Seven districts including Jiangan, Jianghan, Qiaokou, Hanyang, Wuchang, Qingshan, Hongshan) and far from downtown areas (Six districts including Dongxihu, Hannan, Caidian, Jiangxia, Huangpi, Xinzhou). Although the population and medical resources are more concentrated in the central city, we have established a TB service system covering near and far downtown areas [14]. Each district has a TB designated medical institution, including 2 municipal TB designated hospitals.

Household identity was divided into local and migrant. Migrants in China are commonly members of a mobile population who live and work outside the place of household registration, and do not acquired local household identity through the Chinese Hukou system [15]. According to the guidelines for the implementation of China’s tuberculosis prevention and control plan (2008 edition) [16], way of discovering patients was classifies into seven categories:

1. Health examination: TB patients were detected during health check-ups at medical examination facilities.​
2. Contact examination: TB contacts were identified through a known TB case. Contacts can be household members, individuals residing in the same institution, workmates, or people belong to a wider social network of the TB case.
3. Clinic visit due to symptoms: ​patients with suspected TB symptoms seek care at TB-designated medical facilities.​
4. Recommended due to symptoms: patients with suspected TB symptoms were referred by medical staff to TB-designated medical facilities for examination.​
5. Referral: TB patients seeking care at non-TB facilities were transferred to TB facilities when X-rays and sputum smears were suggestive of TB.​
6. Tracing: TB patients and suspected TB patients, who have been transferred by medical staff but failed to attend the TB facility, were followed up to make them go to the designated medical institutions for examination.
7. Others: in addition to the above 6 ways.

Patient delay was defined as the time elapsed from the onset of symptoms to the first medical visit. LPD defined as patient delay longer than 14 days based on the 2004 Tuberculosis Control and Assessment Protocol sponsored by the Chinese Ministry of Health and Center for Disease Control and Prevention [17].

### Statistical analysis

Continuous variables were presented as mean (Standard deviation, SD) or media (Interquartile Range, IQR), and categorical variables were presented as number (percentages). We used ${\chi ^2}$ tests for categorical variables. Independent associations of area and household identity with LPD, as well their interaction effect, were tested by logistic regression models, and we observed a significant area × household identity interaction (*P* < 0.001). Because age was found to significantly modify the relation between household identity and LPD among patients in different area, stratified analysis by area was employed to further evaluated age and household identity on the risk of LPD. In multivariate logistic regression models, age was introduced as a categorical variable. The interaction between age group and household identity was investigated using eight subgroups: age group (< 25 years) * local (reference), age group (25–44 years) * local, age group (45–64 years) * local, age group (≥ 65 years) * local, age group (< 25 years) * migrant, age group (25–44 years) * migrant, age group (45–64 years) * migrant, age group (≥ 65 years) * migrant. Multivariate models were adjusted for gender, occupation, way of patients discovering, treatment history, and sputum smear status. Statistical analyses were performed by using SAS 9.4 for Windows (SAS Institute Inc., Cary, NC, USA). In the two-tailed tests, a *P* value < 0.05 was considered statistically significant.

## Results

### General characteristics of the pulmonary TB patients

Out of the total of 63,720 pulmonary TB patients, 45,407 (71.3%) were males, 22,202 (34.8%) were in the 45–64 age group, 30,163 (47.0%) living far from downtown, 53,528 (84.0%) were locals, 17,297 (27.2%) were unemployed, 37,009 (58.1%) were discovered by referral, 3709 (5.8%) were previously treated, and 28,613 (44.9%) had a positive sputum smear (Table 1).

**Table 1:** Characteristics of pulmonary TB patients and the proportion of long patient delay in Wuhan, China, 2008–2017

Variables			Patient delay		
		Total	≤ 14 days	> 14 days	*P* value
**Gender**					0.746
	Male	45,407 (71.3)	26,641 (58.7)	18,766 (41.3)	
	Female	18,313 (28.7)	10,719 (58.5)	7594 (41.5)	
**Age (years)**					< 0.001
	< 25	12,187 (19.1)	7567 (62.1)	4620 (37.9)	
	25–44	17,696 (27.8)	10,670 (60.3)	7026 (39.7)	
	45–64	22,202 (34.8)	12,678 (57.1)	9524 (42.9)	
	≥ 65	11,635 (18.3)	6445 (55.4)	5190 (44.6)	
**Area**					< 0.001
	Near downtown	34 045 (53.0)	21,544 (63.4)	12,426 (36.6)	
	Far from downtown	30 163 (47.0)	15,816 (53.2)	13,934 (46.8)	
**Household identity**					< 0.001
	Local	53,528 (84.0)	30,741 (57.4)	22,787 (42.6)	
	Migrant	10,192 (16.0)	6619 (64.9)	3573 (35.1)	
**Occupation**					< 0.001
	Service	2513 (3.9)	1627 (64.7)	886 (35.3)	
	Student	5345 (8.4)	3377 (63.2)	1968 (36.8)	
	Worker	4038 (6.3)	2386 (59.1)	1652 (40.9)	
	Farmer	14,612 (22.9)	7819 (53.5)	6793 (46.5)	
	Official staff	2402 (3.8)	1416 (59.0)	986 (41.1)	
	Retired staff	7009 (11.0)	4243 (60.5)	2766 (39.5)	
	Unemployment	17,297 (27.2)	10,275 (59.4)	7022 (40.6)	
	Other	10,504 (16.5)	6217 (59.2)	4287 (40.8)	
**Way of discovering patients**					< 0.001
	Health examination	457 (0.7)	380 (83.2)	77 (16.9)	
	Contact examination	43 (0.1)	31 (72.1)	12 (27.9)	
	Clinic visit due to symptoms	12,723 (20.0)	7937 (62.4)	4786 (37.6)	
	Recommended due to symptoms	127 (0.2)	57 (44.9)	70 (55.1)	
	Referral	37,009 (58.1)	22,236 (60.1)	14,773 (39.9)	
	Tracing	12,539 (19.7)	6109 (48.7)	6430 (51.3)	
	Other	822 (1.3)	610 (74.2)	212 (25.8)	
**Treatment history**					0.037
	New	60,011 (94.2)	35,246 (58.7)	24,765 (41.3)	
	Previously treated	3709 (5.8)	2114 (57.0)	1595 (43.0)	
**Sputum smear status**					< 0.001
	Negative	35,107 (55.1)	20,871 (59.5)	14,236 (40.6)	
	Positive	28,613 (44.9)	16,489 (57.6)	12,124 (42.4)	

Data are presented as the number (percentages)

Data were compared using ${\chi ^2}$ tests

### Patient delay among pulmonary TB patients

The median patient delay was 10 days (IQR, 3–28), and the mean delay was 23 ± 37 days. A total of 26,360 (41.3%) patients’ delay time were longer than 14 days. The proportion of LPD increased with age, from 37.9% among patients in < 25 years to 44.6% among patients aged ≥ 65 years, and it was higher among patients living far from downtown than patients living near downtown (46.8% vs. 36.6%) and higher among locals than migrants (42.6% vs. 35.1%). In addition, the retreated patients had greater proportion of LPD than the newly diagnosed patients (43.0% vs. 41.3%), and sputum smear-positive patients were more likely to have LPD than sputum smear-negative patients (42.4% vs. 40.6%). However, there was not statistically significantly different between males and females (Table 1).

### Trends of LPD among pulmonary TB patients from 2008 to 2017

**Fig. 1:**
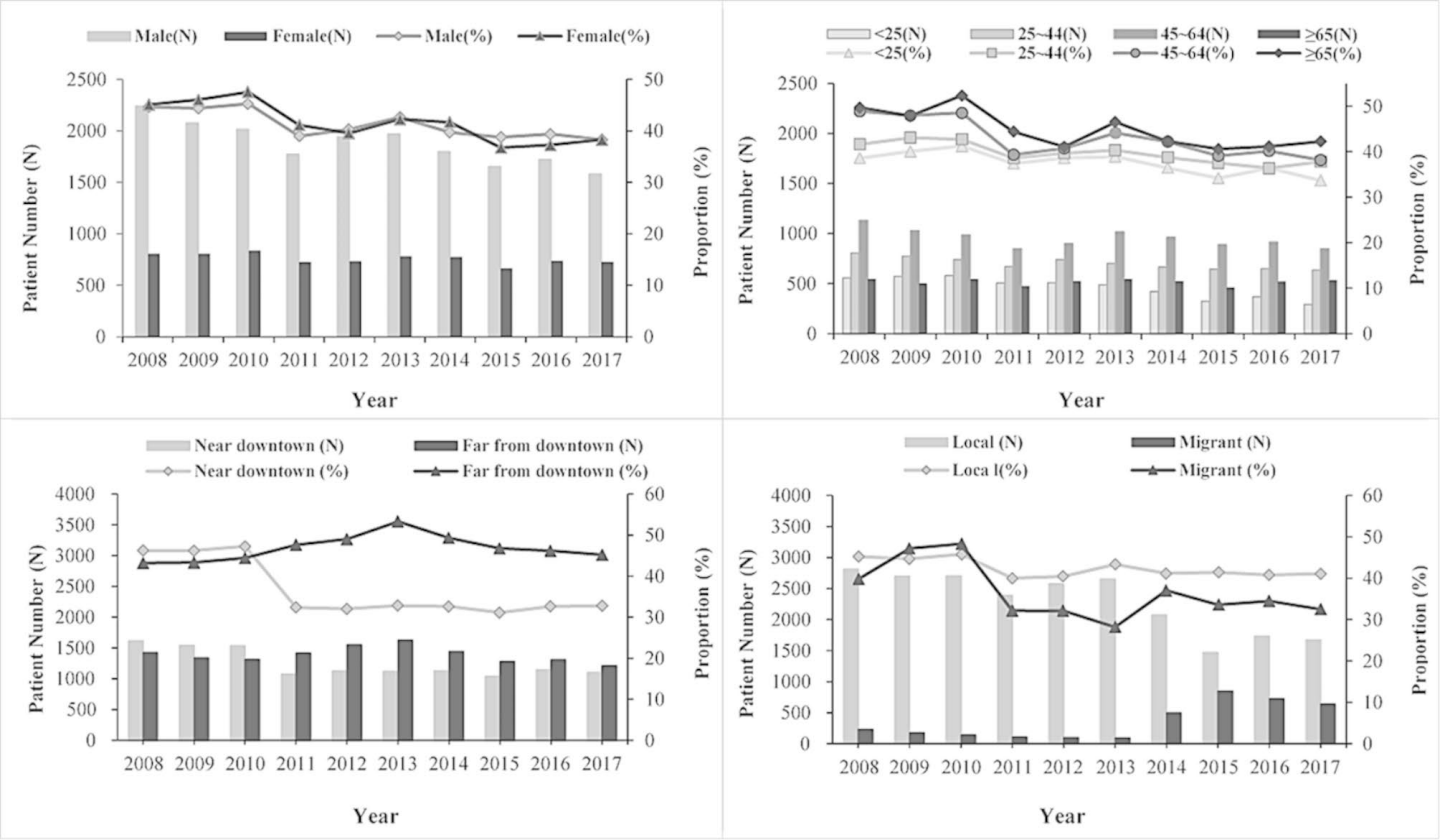
The trend of long patient delay among pulmonary TB patients in Wuhan, China, 2008–2017 Chi-square trend test was used for trend analysis, all *P* value < 0.001

The proportion of LPD decreased from 44.8% in 2008 to 38.3% in 2017, and the decline was more obvious after 2011. Similar trends were also observed in subgroups by gender, age and household identity. The proportion of LPD decreased from 44.7 to 38.3% in males, 45.1–38.3% in females, 38.6–33.7% in < 25 age group, 41.6–37.8% in 25–44 age group, 48.9–38.2% in 45–64 age group, and 49.7–42.3% in ≥ 65 age group, 45.2–41.1% in locals, and 39.8–32.5% in migrants, respectively. Although the proportion of LPD in patients living near downtown decreased from 46.3 to 32.8%, a converse trend was observed for patients living far from downtown, from 43.2 to 45.2% (${\chi ^2}$ test for trends, all *P* < 0.001, Fig. 1).

### Interaction of age and household identity with LPD among pulmonary TB patients in different area

The interaction between age and household identity, and age and area on LPD were significant, after adjusting for gender, age, area, household identity, occupation, way of patients discovering, treatment history and sputum smear status.

**Fig. 2:**
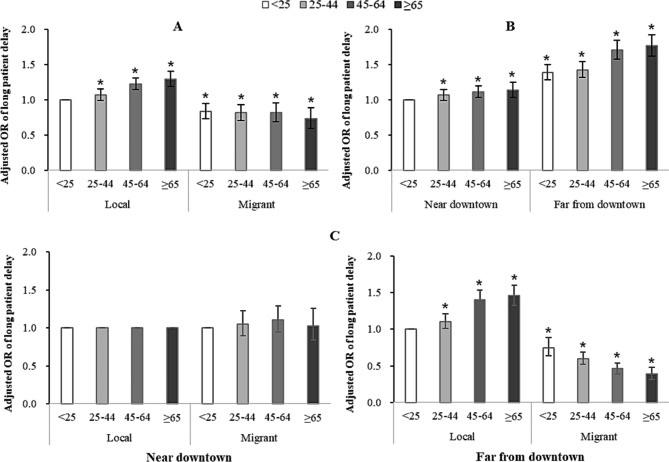
**(A**) Interaction of age and household identity on the risk of long patient delay. **(B)** Interaction of age and area on the risk of long patient delay. **(C)** Interaction of age and household identity on the risk of long patient delay stratified by area. Error bars indicate represent 95% confidence intervals. ^*^P<0.001. Data were analyzed using multivariate logistic regression after adjustment for gender, age, area, household identity, occupation, way of patients discovering, treatment history and sputum smear status

Further evaluation on the effects of age and household identity on LPD stratified by area showed that among patients living far from downtown, the risk of LPD increased with age in local patients, while decreased with age in migrant patients, and the adjust odds ratios (ORs) and 95% confidence interval (CI) of the interaction between age and household identity were as follows: 1.104 (1.009–1.208), 1.403(1.284–1.533) and 1.457 (1.324–1.603) for local patients aged 25–44 years, 45–64 years and ≥ 65 years, respectively, and 0.749 (0.635–0.883), 0.596 (0.517–0.686), 0.459 (0.392–0.538) and 0.387 (0.315–0.476) for migrant patients aged < 25 years, 25–44 years, 45–64 years and ≥ 65 years, respectively. No significant relationship between household identity and age on LPD was observed in patients living near downtown (Fig. 2).

## Discussion

Our study described the 10-year trend of pulmonary TB patient delay and identified several vulnerable groups that might be ignored during fast aging and urbanization. The overall median patient delay in our study was 10 days, which was equivalent to other Chinese cities [18], and much lower than that in African or European countries [19–21]. In the decade from 2008 to 2017, the proportion of LPD among pulmonary TB patients in Wuhan reduced from 44.8 to 38.3%. However, the extent of reduction varied among subgroups. The proportion of LPD decreased more among patients who were migrants or living near downtown. Among patients living far from downtown, the risk of LPD increased with age in local patients, while decreased with age in migrant patients. These findings fill the gap in research on evolutionary path of LPD in the context of rapid aging and urbanization, and identify the vulnerable groups during this process.

Many studies evaluating trends in diagnosis delay and treatment delay among TB patients have actually been reported [8, 22, 23]. However, few studies have examined trends in patient delays, and a study in Portugal found that health delays remained relatively constant while patient delays increased [24]. Our study found that the proportion of LPD has declined over the past 10 years, and a remarkable decline of LPD was detected in 2011. This might be explained by the TB service integration initiative in China. To improve the accessibility and continuity of TB services, the cooperation mechanism of TB prevention institutions and hospitals was reengineered. TB patients’ diagnosis and treatment were provided by the CDC in the early stage, which also provides outpatient services and public health services. To address the shortage of CDC staff and insufficient capacity for diagnosis and treatment, China began a new service delivery model in 2011 that transferred the function of TB diagnosis and treatment from the CDC to TB designated hospitals [14]. After the reform, the TB prevention institutions were more focused on patient management, such as patient screening, tracing and follow-up, while the hospitals were entrusted clearer responsibilities in patient detection, diagnosis and treatment [25]. Meanwhile, joint efforts, including community education, referral notification and high-risk population screening, were also made to improve early TB case detection.

Aging has been proved an important factor leading to LPD, both at the individual level and social level [11, 26]. Elderly populations often suffer from chronic comorbidities, and are more likely to be in bad financial statues and lack of TB knowledge, which may hinder their early service seeking [27, 28]. Wuhan’s population has been aging rapidly in the past decade, thus made the timely finding of geriatric TB patients one of the major challenges. In response, the local government integrated health screening for the elderly into the local TB control package since 2009. In our study, although the overall proportion of LPD still increased with age, the biggest ten-year reduction was detected in ≥ 65 age group (from 49.7 to 42.3%). This indicates health screening for the elderly might be an effective strategy to reduce the LPD in elderly population.

Migration and living in remote areas are increasingly recognized as LPD risk factors with rapid urbanization around the world [29]. Migrants, especially those from high burden areas, often have low economic access to medical services due to poor economic and medical security status [30]. Furthermore, young migrants turned to overestimate their health statues and delayed in seeking medical care [31]. Similarly, patients living in in rural areas or low-resource settings often have poor access to TB services because of their poor economic status or far distance from medical institutions [9, 12, 32−35]. Our study shows that approximately 47% TB patients living far from downtown and had higher odds of LPD in Wuhan. However, the proportion of LPD in migrant patients was lower than that in local patients (35.1% vs.42.6%), and in addition, with a more significant 10-year reduction (39.8–32.5% in migrant patients, and 45.2–41.1% in local patients).

To further clarify the specific role of aging, household identity and living area, we applied interaction effect analysis among these factors on LPD. We found that the proportion of LPD of patients living near downtown was significantly lower than patients living far from downtown, among whom the local residents, especially middle-aged and elderly residents, are much more likely to experience longer delay. In contrast, young migrants living far from downtown had the highest LPD risk among all migrant subgroups. Thus highlighted two vulnerable groups to LPD in Wuhan, the elderly local and young migrant patients living far from downtown.

The government’s TB prevention and employment policies, along with social and cultural status differences among these subgroups, may provide a good explanation to this phenomenon. Chinese government has placed great emphasis on TB detection among migrants and provides free chest X-rays and anti-tuberculosis drugs for all TB patients. Meanwhile, migrants need to provide health certificates, in which chest imaging examination was included, to get a formal job in Wuhan. In addition, migrant population working in big cities tend to be younger, better educated and have better health literacy, compared with the local elderly living far from downtown. This might indicate that while aging and urbanization draw the government’s attention to some certain groups, other routinely considered low-risk groups are being ignored.

The findings of this study have to be seen in light of some limitations. First, our data were based on TBIMS, which collected TB patients’ personal main information on the detection, diagnosis and treatment. We did not obtain full information, such as education, monthly household income, medical insurance, and TB knowledge, which have been proven to be associated with LPD in previous studies [36–38]. Second, there could be a recall bias, that is, the date of onset of symptoms is reported by the patient himself. This may lead to over- or underestimation of the delay time of patient access to health care. However, TB cases were evaluated by a physician through face-to-face consultation, and asked detail about the date of onset of symptoms and the date of first medical visit. Therefore, the collected information was likely to be accurate. In addition, previous studies have found that it was unlikely to affect the overall accuracy of results because it involved all subsets of patients [39]. Third, the study enrolled only patients in Wuhan, limiting the generalizability of findings to other geographic regions. Forth, the age categorization in the study was based on the distribution of the data which may limit its comparability with other studies.​.

## Conclusion

Although the overall LPD among pulmonary TB patients declined in the past decade, the extent of reduction varied in different subgroups. While aging and urbanization draw the government’s attention to some certain groups, other routinely considered low-risk groups are being ignored. The elderly local and young migrant patients living far from downtown are the most vulnerable groups to LPD in Wuhan. Policymakers of TB program should focus on the combined effects of population aging and urbanization on the challenges of TB control. New vulnerable populations, such as elderly local and young migrant living far from downtown, might became a source of health inequities during the process.

## Electronic supplementary material

Below is the link to the electronic supplementary material.

Supplementary Material 1

## Data Availability

The data that support the findings of this study are available from TBIMS but restrictions apply to the availability of these data, which were used under license for the current study, and so are not publicly available. Data are however available from the authors upon reasonable request.

## Abbreviations

TB: Tuberculosis
LPD: Long patient delay
TBIMS: Tuberculosis Information Management System
SD: Standard deviation
IQR: Interquartile Range
ORs: Odds ratio
CI: Confidence interval.
