# Supplementary material for: Trends of a decade in risk factors of patient delay among pulmonary tuberculosis patients during fast aging and urbanization - analysis of surveillance data from 2008 to 2017 in Wuhan, China
Source: BMC Public Health. 2023 May 2;23:803. doi: 10.1186/s12889-023-15707-7 (PMC10155439; doi:10.1186/s12889-023-15707-7)
Supplement: Supplementary file 1 — Supplementary Material 1 [file 12889_2023_15707_MOESM1_ESM.docx]

**Supplemental results**

**Comparison between Included and Excluded Pulmonary TB Participants**

A total of 64287 TB patients were registered at Wuhan TBIMS from January 2008 to December 2017, 489 patients were extrapulmonary TB, and 63798 patients were pulmonary TB. TB patients (78, 0.1%) were excluded from the study due to missing date of onset of symptoms or the first medical visit, and eventually 63720 pulmonary TB patients were included. The distribution of age and area were not statistically significantly different between included and excluded pulmonary TB patients (both *P*>0.05), while there were significant differences in the gender distribution (*P*<0.05, Table S2).

| **Table S1** Basic condition of Wuhan, China^*^ | | | | | | | | | | | |  |
| --- | --- | --- | --- | --- | --- | --- | --- | --- | --- | --- | --- | --- |
| Year | Resident Population (10000 persons) | Registered Population (10000 Persons) | ≥60 years (%) | Urban Population (%) | GDP (Billion) | GDP per capita (Yuan） | Health technicians (Person) | Beds (Bed) | Number of Beds per 1000 Population | Number of Medical Personnel per 1000 Population | Number of Health Technical Personnel per 1000 Population | |
| 2017 | 1089.29 | 853.65 | 20.95 | 72.6 | 13090.81 | 120880 | 102524 | 91635 | 8.41 | 11.70 | 9.41 | |
| 2016 | 1076.62 | 833.85 | 20.72 | 71.7 | 11531.42 | 107902 | 98316 | 87408 | 8.12 | 11.42 | 9.13 | |
| 2015 | 1060.77 | 829.27 | 19.75 | 70.6 | 10547.67 | 100714 | 91763 | 80726 | 7.61 | 10.78 | 8.65 | |
| 2014 | 1033.80 | 827.31 | 18.86 | 67.6 | 10025.93 | 97538 | 78492 | 72827 | 8.85 | 11.83 | 9.54 | |
| 2013 | 1022.00 | 822.05 | 15.62 | 67.6 | 8747.64 | 86014 | 72164 | 66563 | 7.12 | 11.02 | 8.78 | |
| 2012 | 1014.00 | 821.71 | 16.71 | 67.5 | 7752.52 | 76986 | 68253 | 62178 | 7.07 | 10.41 | 8.31 | |
| 2011 | 1004.00 | 827.24 | 15.96 | 66.1 | 6586.52 | 66512 | 64650 | 56540 | 6.51 | 9.86 | 7.82 | |
| 2010 | 978.54 | 836.73 | 15.23 | 65.1 | 5458.35 | 57805 | 62009 | 51199 | 6.10 | 9.50 | 7.41 | |
| 2009 | 910.00 | 835.55 | 14.58 | 65.0 | 4741.69 | 52481 | 60038 | 48061 | 5.80 | 8.90 | 7.20 | |
| 2008 | 897.00 | 833.24 | 13.77 | 64.5 | 4064.62 | 45466 | 57651 | 43435 | 5.20 | 8.60 | 6.90 | |

*Data were from “Wuhan Statistical Yearbook 2009-2018”.

| **Table S2** Comparison between Included and Excluded Pulmonary TB patients | | | | | | |
| --- | --- | --- | --- | --- | --- | --- |
| Variables | | TB Patients | Extrapulmonary TB Patients | Pulmonary TB Patients | | |
|  |  |  |  | Included | Excluded | *P* value |
| Total | | 64287(100.0) | 489 (0.8) | 63720 (99.1) | 78 (0.1) |  |
| Gender | |  |  |  |  | 0.004 |
|  | Male | 45783 (71.2） | 309 (63.2） | 45407 (71.3) | 67 (85.9） |  |
|  | Female | 18504 (28.8） | 180 (36.8） | 18313 (28.7) | 11 (14.1） |  |
| Age (years) | |  |  |  |  | 0.055 |
|  | 0-24 | 12336 (19.2） | 141 (28.8） | 12187 (19.1) | 8 (10.3） |  |
|  | 25-44 | 17859 (27.8） | 139 (28.4） | 17696 (27.8) | 24 (30.8） |  |
|  | 45-64 | 22386 (34.8） | 148 (30.3） | 22202 (34.8) | 36 (46.1） |  |
|  | ≥65 | 11706 (18.2） | 61 (12.5） | 11635 (18.3) | 10 (12.8） |  |
| Area | |  |  |  |  | 0.135 |
|  | Near downtown | 34081 (53.0） | 273 (55.8） | 34045 (53.0) | 43 (55.1） |  |
|  | Far from downtown | 30206 (47.0） | 216 (44.2） | 30163 (47.0) | 35 (44.9） |  |
| Data are presented as the number (percentages).  Data were compared using χ^2^ tests. | | | | | | |

| **Table S3**  Population by District in Wuhan | | | | | |
| --- | --- | --- | --- | --- | --- |
| District | Land Area  ( sq. km) | Population Density (person/sq. km) | | Permanent Population by District (10000 person) | |
|  |  | 2008 | 2017 | 2008 | 2017 |
| Wuhan | 8494.41 | 1056 | 1271 | 897.0 | 1089.3 |
| Near downtown |  |  |  |  |  |
| Jiangan | 64.24 | 11768 | 11988 | 75.6 | 96.2 |
| Jianghan | 33.43 | 19862 | 25790 | 66.4 | 73.0 |
| Qiaokou | 46.39 | 15628 | 21679 | 72.5 | 86.9 |
| Hanyang | 108.34 | 6277 | 5898 | 68.0 | 65.3 |
| Wuchang | 81.22 | 14504 | 19793 | 117.8 | 127.6 |
| Qingshan | 45.80 | 11026 | 9349 | 50.5 | 52.9 |
| Hongshan | 509.00 | 2206 | 2851 | 112.3 | 163.8 |
| Far from downtown | |  |  |  |  |
| Dongxihu | 439.19 | 856 | 1133 | 37.6 | 56.3 |
| Hannan | 287.70 | 379 | 467 | 10.9 | 13.6 |
| Caidian | 1108.10 | 388 | 667 | 43.0 | 73.5 |
| Jiangxia | 2010.00 | 315 | 453 | 63.4 | 91.4 |
| Huangpi | 2261.00 | 408 | 437 | 92.2 | 98.8 |
| Xinzhou | 1500.00 | 579 | 616 | 86.8 | 90.2 |


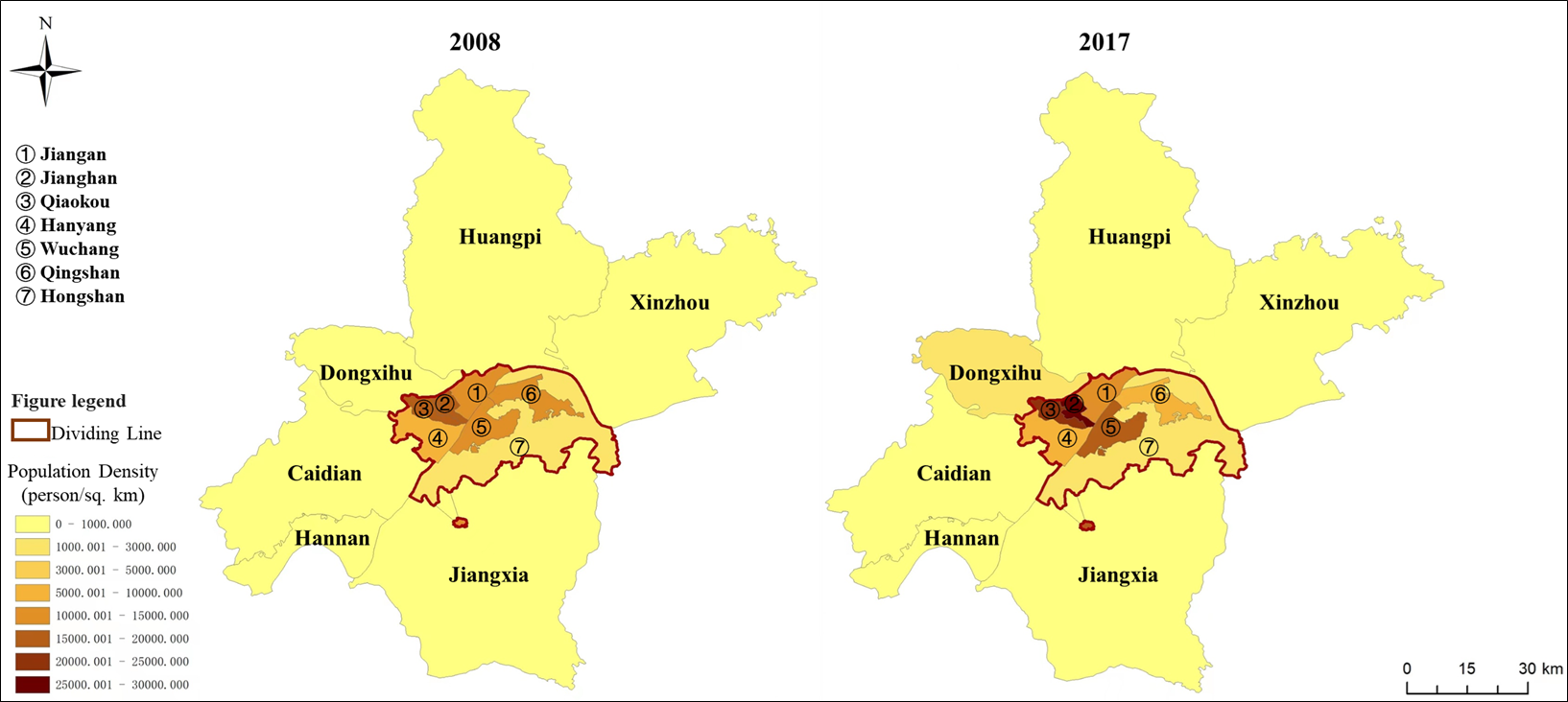


**Figure S1** Population Density of Wuhan (Person/sq. km)
